# Behavioral and Cerebral Impairments Associated with Binge Drinking in Youth: A Critical Review

**DOI:** 10.5334/pb.476

**Published:** 2019-03-29

**Authors:** Séverine Lannoy, Joël Billieux, Valérie Dormal, Pierre Maurage

**Affiliations:** 1 Cognition Health Society Laboratory (C2S – EA 6291), Université de Reims Champagne-Ardenne, Reims, FR; 2 Laboratory for Experimental Psychopathology (LEP), Psychological Science Research Institute, Université catholique de Louvain, Louvain-la-Neuve, BE; 3 Addictive and Compulsive Behaviours Lab (ACB-Lab), Institute for Health and Behaviour, University of Luxembourg, Esch-sur-Alzette, LU

**Keywords:** binge drinking, neuropsychology, electrophysiology, neuroimaging, alcohol-use disorders, alcohol use disorder

## Abstract

Binge drinking is a widespread alcohol consumption pattern in youth that is linked to important behavioral and cerebral impairments, in both the short and the long term. From a critical review of the current literature on this topic, we conclude that binge drinkers display executive impairments, cerebral modifications, and problems with emotion-related processes. Five key empirical and theoretical topics are discussed to pave the way for future research in the field: (1) the specificity of the brain modifications observed in binge drinkers that may index a compensatory mechanism or result from multiple withdrawals; (2) the nature of the relationship between binge drinking and impairments, suggesting reciprocal influences between excessive alcohol consumption and executive deficits; (3) the possible recovery of brain and cognitive functioning after the cessation of binge drinking; (4) the validity of the continuum hypothesis, suggesting links between binge drinking and severe alcohol use disorders; and (5) the existing strategies to reduce binge drinking habits or rehabilitate the associated cognitive deficits. Future perspectives are described in relation to the questions raised to identify the crucial variables to be addressed in research and clinical practice.

## Introduction

Alcohol consumption is widespread in Western societies and occurs in many everyday life situations. It also occupies an important position among young people, particularly among students, where it is a key component of the so-called academic folklore (Wechsler & Nelson 2001). Despite this ubiquity of alcohol consumption, leading most people to consider it a relatively harmless substance, the danger of excessive drinking habits has been recognized. Alcohol-related disorders, currently identified as the third risk factor for premature death by the World Health Organization (WHO), lead to 3 million deaths per year worldwide (WHO, 2016). Excessive alcohol consumption is thus considered a central public health problem (Nutt, King, & Phillips 2010). The consequences of alcohol abuse and dependence have also been extensively explored in scientific research, which has mainly focused on long-term and chronic alcohol-related problems and shown, for example, cognitive (Stavro, Pelletier & Potvin 2013), emotional (Donadon & Osório 2014), interpersonal (Thoma, Friedmann & Suchan 2013), and cerebral (Bühler & Mann 2011) impairments related to alcohol dependence (as it is referred to in the DSM-IV) or severe alcohol use disorders (as it is referred to in the DSM-5). Aside from these consequences in severe alcohol use disorders, which have been largely documented, growing interest has recently been observed in the effects of alcohol consumption on non-alcohol-dependent young people. Because nearly 2000 deaths per year in the United States are related to excessive drinking in students (Hingson, Zha & Weitzman 2009), research has started to explore whether excessive drinking leads to deleterious consequences and, if so, at which consumption threshold and frequency (Hingson, Zha, & White, 2017). Notably, some studies have suggested that the specific binge drinking pattern, characterized by repeated alternations between intense intoxication and withdrawal, could be associated with cognitive and emotional impairments (Stephens & Duka 2008).

The problem of binge drinking is widespread in youth: 40% of adolescents and young adults in Western countries presently report at least one binge drinking episode per month (e.g., Archie et al., 2012; Kanny et al., 2013). Other studies have found that this consumption pattern leads to cognitive and cerebral consequences (Hermens et al., 2013; Maurage, Petit & Campanella 2013a). It has even been suggested that binge drinking could be considered a first step toward severe alcohol use disorders (Bonomo et al., 2004). The main aim of this paper is therefore to identify the specific behavioral and cerebral impairments related to binge drinking habits in young adults through a critical review. It is focused on two main processes that have been identified as central to the understanding of alcohol-related disorders: executive functions and emotional processing. Beyond this literature review, we also address five specific research avenues: (1) the specificity of the brain modifications observed in binge drinkers, (2) the nature of the relationship between binge drinking and behavioral/cerebral impairments, (3) the possible persistence of these impairments after the cessation of binge drinking habits, (4) the continuum hypothesis that links binge drinking with severe alcohol use disorders, and (5) the perspectives for prevention and intervention programs in binge drinking. These research avenues have been identified on the basis of major perspectives or limitations underlined in the existing literature. It is therefore essential to offer a critical state-of-the-art discussion about these currently prominent issues related to the binge drinking research field.

### Definition and Conceptualization of Binge Drinking

Binge drinking refers to an excessive but episodic alcohol consumption pattern, characterized by repeated alternations between large alcohol intakes and periods of abstinence. Nevertheless, to date, the conceptualization of binge drinking remains a matter of debate in the scientific literature, and no consensus has been reached regarding its definition. Two main proposals can, however, be identified in existing studies that target alcohol consumption (i.e., number of drinks consumed) or specific drinking pattern (i.e., computation of a binge drinking score).

The first classification that was proposed to capture binge drinking is based on the number of drinks consumed on one occasion, commonly recognized as the 5/4 measure (i.e., 5 alcohol units for men, 4 for women; Wechsler & Nelson 2001; Labhart et al., 2018). Such an approach has been widely used in epidemiological and public health research (e.g., Kanny et al., 2013). This proposal was further specified through the calculation of the blood alcohol concentration (BAC) level. Specifically, the National Institute on Alcohol Abuse and Alcoholism (NIAAA, 2004) describes binge drinking as an alcohol consumption pattern leading to a BAC level of 0.08 g/dL, typically observed after four (for women) or five (for men) alcohol drinks over a period of 2 hours. To be considered binge drinkers, individuals have to present such a consumption pattern at least once a month. Although focusing on the BAC level offers an initial objective definition, these quantities should take into account national differences (e.g., an alcohol unit contains 8 g of pure ethanol in the United Kingdom, whereas it contains 10 g in most European countries and 14 g in the United States). Another potential shortcoming of this definition is that it does not focus on the frequency of drinking habits, which is known to be a key variable in explaining the emergence of neurological consequences and alcohol use disorder.

Second, some authors have suggested that binge drinking should be described in reference to the drinking pattern rather than to alcohol consumption per se (Townshend & Duka 2002). This approach appears to be particularly well adapted to the exploration of drinking among people who consume alcohol in large quantities but in an irregular pattern (i.e., binge drinkers), which might not be detected with the NIAAA definition. The computation of a binge drinking score was thus proposed, based on (1) consumption speed (i.e., number of alcohol units consumed per hour), (2) number of drunkenness episodes (drunkenness being defined as reduced ability to speak clearly, loss of coordination, and nausea), and (3) percentage of drunkenness episodes (i.e., ratio of drunkenness episodes to the total number of drinking episodes) in the last 6 months. More precisely, the following formula has been proposed to compute a binge drinking score: [(4 × consumption speed) + number of drunkenness episodes + (0.2 × percentage of drunkenness episodes)]. This binge drinking score has been widely used in the literature (e.g., Bø et al., 2016a; Czapla et al., 2015; Kessler et al., 2013) and cutoff scores have been provided to differentiate non-binge drinkers from binge drinkers (Townshend & Duka 2005). However, these cutoff scores also depend on the quantity of alcohol contained in one unit. Typically, the initial studies that used this binge drinking score (Townshend & Duka 2002, 2005) were conducted in England, where an alcohol unit contains 8 g of pure ethanol. In these studies, a score higher than 23 indicated intense binge drinking, whereas a score lower than 17 characterized non-binge drinkers. In Belgium, where an alcohol unit comprises 10 g of pure ethanol, adapted cutoffs have been proposed in accordance with other alcohol variables, such as number of doses consumed per occasion or consumption speed (e.g., Lannoy et al., 2017a): binge drinkers were thus defined as having a score higher than 17, whereas non-binge drinkers have a score lower than 13. Another advantage of the binge drinking score is that it indirectly takes into account enhancement-related motives (e.g., euphoria), which constitute strong predictors of binge drinking (e.g., Kuntsche et al., 2014). Consideration of consumption speed and drunkenness allows one to target the motivational aspects of drinking (e.g., drinking a large amount to rapidly become intoxicated and thus feel the positive sensations associated with alcohol).

### Definition of Related Alcohol Consumption Patterns

In this section, we briefly describe the different terms used to refer to excessive drinking among young adults that may be related to binge drinking, including *hazardous drinking, heavy episodic drinking*, and *social drinking*. Some of the studies that we review focus on these alcohol consumption patterns and describe them with the number of “binge drinking episodes” in a specific time frame, thus proposing a direct link with binge drinking.

According to the WHO, alcohol consumption ranges from hazardous drinking to physical dependence (Babor & Higgins-Biddle, 2001). *Hazardous drinking* refers to a repetitive pattern of alcohol consumption that leads to physical, mental, and social consequences. This drinking mode consists of the consumption of at least five (women) or seven (men) drinks per occasion at least three times per week. In the literature, the Alcohol Use Disorders Identification Test score is used to categorize individuals as hazardous drinkers (e.g., Palfai & Ostafin, 2003; Van Tyne et al., 2012), typically when they have a score higher than seven.

*Heavy episodic drinking* is defined by the NIAAA as a binge drinking pattern (at least four or five United States alcohol units consumed per occasion) that occurs more than once a week (NIAAA, 2004). In most studies, heavy drinking is used as a synonym for binge drinking, without necessarily representing a more frequent pattern of excessive drinking (e.g., Nederkoorn et al., 2009).

*Social drinking* is mainly related to the motivations underlying alcohol consumption and characterizes individuals who drink alcohol in social contexts. However, in the literature, this term is often used to refer to excessive drinkers (mostly according to weekly alcohol consumption, e.g., Townshend & Duka, 2001) but without a systematic evaluation of drinking motives or alcohol expectancies (Petit et al., 2012).

### Brief Conclusion

This section highlighted the various related concepts used to define excessive alcohol consumption habits in youth, particularly binge drinking. Most studies that focus on binge drinking use one definition or another, but combining these definitions to simultaneously consider the BAC level and the binge drinking score might lead to a more valid definition of specific binge drinking habits. However, such a definition has to be considered in terms of large samples and different populations so that a better consensus can be proposed regarding its use. Despite this ongoing debate about definitions, many studies have explored the consequences of binge drinking during the last decades in order to specify the cognitive and cerebral impairments related to this drinking pattern. In the next sections, we first review the behavioral results that show that binge drinking is related to large-scale executive deficits, and then describe the studies that used neuroscience techniques to determine the brain modifications observed among binge drinkers.

## Literature Review

The current literature review capitalizes on a critical approach to cover several important fields in the binge drinking literature before identifying and discussing the major issues in these fields. In particular, we focus on two central processes involved in addiction and even more so in alcohol-related disorders: executive functions and emotional processing. All of the studies that refer to binge drinking are included in this review, even though the definition of binge drinking is distinctly different among them (as mentioned earlier). We did not consider studies that aimed to determine acute alcohol effects or that targeted comorbidity between binge drinking and psychopathological disorders. In addition to considering behavioral results, we also address cerebral modifications related to binge drinking, in particular cerebral changes during cognitive tasks, in order to connect them with the behavioral findings. Moreover, in the neuroscience section, we mainly focus on electrophysiological studies that describe the changes related to specific cognitive stages. Finally, we briefly address neuroimaging data that provide a clear understanding of the compensatory brain mechanisms described in binge drinking (see critical discussion for more details).

### Executive impairments associated with binge drinking

Executive functions have been studied intensively because impairments in these functions are a crucial feature of severe alcohol use disorders (Wilcox et al., 2014). In binge drinking, studies that explored these difficulties showed impairments in flexibility and in inhibition in particular (Table 1).

**Table 1:** Summary of the behavioral studies exploring executive functions in binge drinking.

	Participants	Tasks	Results

**Flexibility**			

Hartley et al. (2004)	Young adults (N = 27; mean age: 21.1 yo) NIAAA criteria Binge drinking score	Intra/Extradimensional Set Shift Task evaluates shifting and attentional flexibility	No group difference
Scaife & Duka (2009)	Young adults (N = 60; mean age: 20.6 yo) Binge drinking score	Intra/Extradimensional Set Shift Task	Female binge drinkers presented poor performance
Salas-Gomez et al. (2016)	Young adults (N = 206; mean age: 19.5 yo) NIAAA criteria	Trail Making Test (A, B) Part B evaluates attentional flexibility	Binge drinkers showed poorer performance
Bø et al. (2017)	Young adults (N = 103; mean age: 21.7 yo) Binge drinking score (continuous approach)	Intra/Extradimensional Set Shift Task	Shifting did not predict the binge drinking score 18 months later
Gil-Hernandez et al. (2017)	Adolescents and young adults (N = 322) categorized according to age (1: 13–15 yo, 2: 16–18 yo, 3: 19–22 yo) NIAAA criteria	Trail Making Test (A, B) Verbal fluency assessing flexibility	Binge drinkers (19–22 yo) presented poorer performance than matched controls in the Trail Making Test
Carbia et al. (2018a)	Adults after 11 years of alcohol consumption (N = 63; age: 29 yo) AUDIT-C score	Trail Making Test, B Verbal fluency	No group difference
**Inhibition**			

Townshend & Duka (2005)	Young adults (N = 100, mean age: 20.9 yo) Binge drinking score	Digit Vigilance Test measures response inhibition during sustained attention	Female binge drinkers showed poorer performance (more commission errors) than female controls
Fernie et al. (2010)	Young adults (N = 75; mean age: 19.3 yo) Total weekly consumption, AUDIT, binge drinking score (continuous approach)	Go/No-Go Task measuring the ability to inhibit an inappropriate response Stop Signal Task measuring the ability to inhibit an already initiated response	Inhibition performance did not predict alcohol use and problems
Goudriaan et al. (2011)	Young adults (N = 200; mean age: 20.4 yo) Consumption of more than 5 drinks at least two times in the past month	Go Stop Task is a combination of Go/No-Go and Stop Signal tasks evaluating prepotent response inhibition	Inhibition performance was not related to alcohol consumption two years later
Sanhueza et al. (2011)	Young people (mean age: 18.9 yo) and elderly adults (mean age: 69.4 yo) [N = 91] For binge drinking, consumption of more than 6 (female) or 8 (male) drinks in two hours	Stroop color-word Task evaluating the ability to avoid an automatic response	Binge drinkers performed worse than controls of similar age. Their performance was comparable to elderly participants in the word reading condition but not in the word naming one.
Moreno et al. (2012)	Young adults (N = 68; mean age: 20 yo) CAGE score, more than 6 drinks in the last drinking episode	Go/No-Go Task Stop Task	No group difference
Henges & Marczinski (2012)	Young adults (N = 109; mean age: 19.6 yo) NIAAA criteria (continuous approach)	Go/No-Go Task	Poor inhibition performance predicted the total consumption in the month, the number of binge drinking episodes, and the highest number of drinks consumed.
Sanchez-Roige et al. (2014)	Young adults (N = 44; mean age: 21.2 yo) Binge drinking score	Stop Signal Task	No group difference
Czapla et al. (2015)	Young adults (N = 32; mean age: 23.8 yo) Binge drinking score	Go/No-Go Task with alcohol-related stimuli	Binge drinkers presented poorer performance in the alcohol condition
Poulton et al. (2016)	Adults (N = 84; mean age: 22.8 yo) Consumption of at least 6 drinks (with more than 2 drinks per hour) at least two times a month	Monetary Incentive Control Task assessing prepotent response inhibition with reward contingency	Binge drinkers presented poorer performance
Salas-Gomez et al. (2016)	Young adults (N = 206; mean age: 19.5 yo) NIAAA criteria	Stroop color-word Task	No group difference
Bø et al. (2016a)	Young adults (N = 121; mean age: 21.7 yo) Binge drinking score (continuous approach)	Stop Signal Task	Binge drinking score did not predict poor inhibition but faster reaction time and less adjustment following commission errors
Lannoy et al. (2018a)	Young adults (N = 44; mean age: 20.9 yo) Consumption of at least 6 drinks (minimum 2 drinks per hour) at least two times a week Binge drinking score	Speeded (time limit to process Go stimuli) Go/No-Go Task with alcohol-related stimuli	Binge drinkers presented poorer adjustment following commission errors in the alcohol condition
**Planning**			

Hartley et al. (2004)	Young adults (N = 27; mean age: 21.1 yo) NIAAA criteria Binge drinking score	Stockings of Cambridge test evaluating planning abilities	Binge drinkers were slower than controls
Sanhueza et al. (2011)	Young people (mean age: 18.9 yo) and elderly adults (mean age: 69.4 yo) [N = 91] For binge drinking, consumption of more than 6 (female) or 8 (male) drinks in two hours	Tower of Hanoi assessing planning abilities	No group difference involving binge drinking
Parada et al. (2012)	Young adults (N = 122; mean age: 18.8 yo) Consumption of at least 6 drinks (minimum 3 drinks/hour) once a month	Zoo Map and Key Search tests measuring planning abilities	No group difference
Mota et al. (2013)	Young adults (N = 89; mean age: 18.7 yo) Consumption of at least 6 drinks (minimum 3 drinks/hour) once a month	Zoo Map and Key Search tests	No group difference at the longitudinal level (after two years of binge drinking)
**Decision-making**			

Goudriaan et al. (2007)	Young adults (N = 200; mean age: 19.9 yo) Consumption of more than 5 drinks at least two times in the past month	Iowa Gambling Task assessing decision making under ambiguity and under risk	At the longitudinal level, the stable high binge drinkers group (according to the frequency) made less advantageous choices in decision making
Xiao et al. (2009)	Adolescents (N = 181; mean age: 16.2 yo) NIAAA criteria	Iowa Gambling Task	Binge drinkers had poorer decision making abilities, and advantageous decision making predicted less drinking problems one year later
Goudriaan et al. (2011)	Young adults (N = 200; mean age: 20.4 yo) Consumption of more than 5 drinks at least two times in the past month	Iowa Gambling Task	Poorer decision making was associated with higher binge drinking two years later
Bø et al. (2016)	Young adults (N = 121; mean age: 21.7 yo) Binge drinking score (continuous approach)	Iowa Gambling Task	Binge drinking score was related to poorer performance in the first trials of decision making
Moreno et al. (2012)	Young adults (N = 68; mean age: 20 yo) CAGE score, more than 6 drinks in the last drinking episode	Iowa Gambling Task Two-Choice Task evaluating decision making	Binge drinkers had poorer decision-making performance

*Note:* The three first processes (flexibility, inhibition, planning) refer to executive functions subcomponents. NIAAA criteria correspond to at least one binge drinking episode per month. Binge drinking score is computed based on the following formula: [(4*consumption speed) + drunkenness frequency + (0.2 * drunkenness percentage)]. AUDIT-C consists of the sum of the three first items of the AUDIT questionnaire assessing alcohol consumption frequency, intensity, and the frequency of binge drinking episodes (more than 6 drinks). CAGE questionnaire evaluates problem drinking by four self-reported items. yo = years old. One drink corresponds to one alcohol unit.

### Flexibility

Concerning flexibility, one study that included people who had at least one binge drinking episode per month required participants to alternately connect randomly presented numbers (in ascending order) and letters (in alphabetical order) in the Trail Making Test (Salas-Gomez et al., 2016). The authors reported that this group had poorer performance in assessments of attention, visuo-spatial abilities, and flexibility. Using the same task and criteria, Gil-Hernandez et al. (2017) specified that this poorer flexibility only appeared in older binge drinkers and not among adolescents. However, another study showed that, when binge drinkers and non-binge drinkers were compared after 11 years, flexibility impairments were not supported by either the Trail Making Test or the verbal fluency task (asking participants to produce as many words as possible in 1 minute that started with a specific letter) (Carbia et al., 2018a). By comparing groups selected on the basis of the binge drinking score, Scaife and Duka (2009) specifically evaluated shifting and attentional flexibility in a task that also involved rule acquisition, visual discrimination, and attentional abilities (Intra/Extradimensional Set Shift Task, which requires participants to learn the characteristics of the correct stimulus, representing color-filled shapes, white lines, or both, while characteristics change across successive blocks). Their findings revealed impaired performance in female binge drinkers (Scaife & Duka 2009). Nevertheless, using the same experimental task but comparing binge drinkers to teetotalers (i.e., participants who never drink alcohol; binge drinkers were recruited according to the NIAAA definition and their binge drinking score), Hartley, Elsabagh, and File (2004) could not identify any impairment in flexibility in binge drinkers. This contradictory result can be explained by the smaller sample size in the study by Hartley et al. (2004) than in that by Scaife and Duka (2009) (14 and 30 binge drinkers, respectively). Nonetheless, although they did not observe flexibility impairments, Hartley et al. (2004) identified group differences in other experimental tasks, suggesting that alternative factors could explain this difference (e.g., related to the control group, as these participants were either totally abstinent or low alcohol consumers in these studies). Finally, a continuous approach that targeted various drinking patterns rather than specific groups showed that shifting did not predict the binge drinking score 18 months later (Bø et al., 2017).

### Inhibition

Inhibition is typically divided into three subcomponents (see Friedman & Miyake 2004): prepotent response inhibition (control of automatic responses), resistance to distractor interference (ability to resist disturbances from inappropriate information), and resistance to proactive interference (ability to resist memory interference from previously relevant information). However, the existing binge drinking literature has mainly focused on prepotent response inhibition, which is more closely related to the control of alcohol consumption (López-Caneda et al., 2014a); the present critical review also focuses on this type of inhibition. Study results have mostly indexed inhibition impairments in binge drinking, which are potentially identified as both the cause and consequence of excessive consumption (López-Caneda et al., 2014a). Moreover, by asking participants to perform a task that also involved sustained attention, one study reported that binge drinkers presented more commission errors than controls did (Townshend & Duka, 2005). Another study showed that in the Stroop color-word task, the performance of binge drinkers was comparable to that of elderly people, suggesting premature aging in binge drinking (Sanhueza, García-Moreno, & Expósito, 2011).

Nevertheless, some studies that used the classic Stroop or Stop Signal tasks did not find impairment of inhibition in binge drinkers (e.g., Salas-Gomez et al., 2016; Sanchez-Roige et al., 2014), suggesting that the involvement of this process is questionable. First, some studies suggested instead that binge drinkers display decision-making deficits (e.g., Moreno et al., 2012). This result is supported by the study of Goudriaan and colleagues (2011), which indicated that decision making, but not prepotent response inhibition, is an efficient predictor of binge drinking habits 2 years later. Similarly, using a continuous approach that aimed to predict binge drinking, some studies found that inhibition difficulties predicted the number of drinks consumed on one occasion (e.g., Henges & Marczinski 2012), while others found that alcohol use and problems in binge drinkers were predicted only by risk-taking tendencies (e.g., Fernie et al., 2010). Second, it has also been argued that binge drinkers present inhibition impairment only during the processing of alcohol-related cues (Czapla et al., 2015). However, with a more complex and reward-based task, binge drinkers had altered inhibition in both neutral and reward contexts (Poulton et al., 2016). Third, some studies indicated that the relationship between binge drinking and inhibition was instead characterized by impairments in performance monitoring, in particular difficulty in adjusting one’s behavioral response following commission errors (Bø et al., 2016a). This proposal was confirmed by using behavioral and electrophysiological measures in case-control studies involving binge drinkers and control participants (Lannoy et al., 2017b; Lannoy et al., 2018a; Smith & Mattick 2013). These inconsistencies between studies were addressed in a recent systematic review of the neuropsychological studies in binge drinking; the authors concluded, after careful consideration of the literature, that binge drinkers are indeed characterized by inhibition impairments (Carbia et al., 2018b).

### Planning

Planning abilities have been evaluated with a task that asks participants to reproduce a specific schema displayed on the upper part of a screen (Stockings of Cambridge test). While there was no difference in the number of moves necessary to obtain the correct schema, binge drinkers were slower than teetotalers in planning their actions, especially at the beginning of the task (Hartley et al., 2004). However, to our knowledge, no study has confirmed these higher level executive difficulties in binge drinkers. Investigators have also stated that the comparison between binge drinkers and teetotalers did not allow them to identify impairments that were specifically related to the consumption pattern, as the difference might be due to a general difference in the quantity of alcohol consumed. In several studies that were based on various experimental paradigms, investigators did not observe planning difficulties in binge drinkers compared with control low drinkers (Parada et al., 2012; Sanhueza et al., 2011), even after 2 years of continuous binge drinking (Mota et al., 2013).

### Decision making

In addition to executive difficulties, disadvantageous choices during decision making have also been identified in binge drinkers compared with controls, mainly through the use of the Iowa Gambling Task (in which participants have to learn the reward probabilities associated with card decks and progressively orient their choices toward long-term rather than short-term rewarding decks). On the basis of alcohol consumption patterns in the past 2 years, high stable binge drinkers (having two or three binge drinking episodes per month for at least 2 years) were compared with the following groups: low binge drinkers (having less than one binge drinking episode per month), stable moderate binge drinkers (having one or two binge drinking episodes per month during the last 2 years), and increasing binge drinkers (low binge drinkers at the first evaluation but having two or three binge drinking episodes per month 2 years later). Results showed that high stable binge drinkers made fewer advantageous choices than did the other groups (Goudriaan, Grekin & Sher 2007). Moreover, decision making also predicted future consumption: disadvantageous choices were associated with higher binge drinking habits 2 years later, mainly in males (Goudriaan, Grekin & Sher 2011), and better choices predicted reduced alcohol consumption and alcohol-related problems 1 year later in adolescents (Xiao et al., 2009). Finally, for a sample of college students, a recent study suggested that the binge drinking score is exclusively related to disadvantageous choices in the first trials of the decision-making task (Bø, Billieux & Landrø 2016b). These decision-making difficulties were identified in binge drinkers with the Iowa Gambling Task, as well as with a two-choice task that required participants to choose between two possibilities: wait 5 seconds and win 5 points or wait 15 seconds and win 15 points (Moreno et al., 2012). Surprisingly, Goudriaan and colleagues (2007) observed that the decision-making performance of binge drinkers was not related to impulsivity (measured with the Impulsive Sensation Seeking Scale and the Barratt Impulsivity Scale), and Moreno and colleagues (2012) showed decision-making impairments in the absence of inhibition difficulties. These results are in line with the proposal of Verdejo-Garcia (2017) that, although decision-making abilities and executive processes may be related, individuals can also have a dissociated pattern characterized by decision-making impairments but preserved executive skills, underscoring the independent contribution of these processes (Verdejo-Garcia, 2017).

### Brief conclusion

In summary, the available data clearly suggest that binge drinking is associated with important and various cognitive impairments, but some findings need to be replicated (e.g., planning difficulties have been observed in only one study). Contradictory results have also been identified, notably regarding inhibition, that might index the implications of specific underlying processes (e.g., performance monitoring). Furthermore, regarding inhibitory control, prepotent response inhibition was mainly considered in these studies, whereas other types of inhibition (i.e., resistance to distractor and to proactive interference) were largely ignored.

## Emotional difficulties associated with binge drinking

Beyond the cognitive impairments observed in using classic neuropsychological paradigms, recent research has highlighted that binge drinking might also be associated with emotional disturbances, although little has been reported in this research field until now. Several studies have described increased depression, anxiety, or negative mood levels in young adult binge drinkers from self-reported measures and have identified negative emotions as vulnerability factors for future binge drinking (e.g., Mushquash et al., 2013; Townshend & Duka 2005). However, these results have not been confirmed by experimental studies that focus on mood induction or affective processing. Moreover, to our knowledge, only seven studies have directly explored the processing of emotional stimuli in binge drinking by means of affective decoding tasks, most of them focusing on neuroscience measures. At the behavioral level, the first study to explore emotional cross-modal integration (i.e., multisensorial decoding of emotions) showed an absence of behavioral differences between binge drinkers and control participants (Lannoy et al., 2017c), but the use of a more complex and sensitive task indexed poorer recognition of emotional facial expressions in binge drinking (Lannoy et al., 2018b). Other findings were observed through neurophysiological and neuroimaging studies. At the electrophysiological level, the event-related potential (ERP) components associated with the processing of emotional human voices (Maurage et al., 2009) and with emotional cross-modal integration (Lannoy et al., 2018c), as well as the images associated with negative valence (Connell, Patton & McKillop, 2015), were disrupted in binge drinkers. Moreover, the affective modulation of event-related theta oscillations was reduced in binge drinkers during the processing of emotional images (Huang et al., 2017). At the neuroimaging level, results showed that young binge drinkers had reduced performance in emotional voice categorization at the behavioral level, linked to a double brain alteration (Maurage et al., 2013b): binge drinkers presented reduced activation in the voice processing area (superior temporal gyrus) but showed increased activation in other areas not usually involved in emotional processing (middle frontal gyrus). Therefore, these preliminary findings suggest that binge drinking might also be associated with impairments in the processing of emotional stimuli. As emotional impairments have been found to play a crucial role in the development and maintenance of severe alcohol use disorders, understanding the early alterations of these emotional difficulties in the first stages of the disorders may be essential and should be investigated further.

## Cerebral modifications associated with binge drinking

### Insights from electrophysiology

To assess what is known about the link between cerebral modifications and behavioral performance, we focus in this section on studies that explored specific ERPs during cognitive and emotional tasks. Alterations of various ERP components have been reported in binge drinkers compared with those in control participants (Table 2), without always being related to detectable behavioral impairments. Cerebral modifications were indexed by either a reduced or an increased amplitude of ERP components.

**Table 2:** Summary of the electrophysiological studies exploring binge drinking.

	Participants	Tasks	Results

**P100 – Visuospatial processing**			

Maurage et al. (2009)	Young adults (N = 36, mean age: 18.2 yo, at first assessment time) Consumption of more than 6 drinks at least once a week	Emotional valence detection task with auditory stimuli	Binge drinkers presented delayed P100 latency after 9 months of binge drinking
Maurage et al. (2012)	Young adults (N = 80, mean age: 21.5 yo) Comparison of low (at least 5 drinks, 2/3 times a week) and high (at least 10 drinks, 3/4 times a week) binge drinking with controls and daily drinkers	Visual Oddball Task	Binge drinkers presented increased P100 latency and a difference was also related to the intensity of binge drinking
Petit et al. (2012)	Young adults (N = 36, mean age: 21.6 yo) NIAAA criteria, consumption of at least 3 drinks/hour	Visual Oddball Task with alcohol-related stimuli	Binge drinkers had higher P100 amplitude during the processing of alcohol-related stimuli
Petit et al. (2014)	Young adults (N = 30, mean age: 22 yo, at first assessment time) Consumption of at least 6 drinks (minimum 3 drinks/hour) at 3 or 4 times a week	Visual Oddball Task with alcohol-related and emotional pictures	Binge drinkers showed reduced P100 amplitude (for all stimuli) after one year
**N100 – Auditory processing/Early processing of unexpected stimulus**			

Maurage et al. (2012)	Young adults (N = 80, mean age: 21.5 yo) Comparison of low (at least 5 drinks, 2/3 times a week) and high (at least 10 drinks, 3/4 times a week) binge drinking with controls and daily drinkers	Visual Oddball Task	Binge drinkers presented increased N100 latency and a difference was also related to the intensity of binge drinking
Watson et al. (2016)	Young adults (N = 50, mean age: 20.6 yo) NIAAA criteria (frequency-based approach to categorize low/high binge drinkers)	Go/No-Go Task with alcohol-related stimuli	High binge drinkers showed larger N100 amplitude than both low binge drinkers and controls (no effect of stimulus type)
**N170 – Visual and face processing**			

Maurage et al. (2012)	Young adults (N = 80, mean age: 21.5 yo) Comparison of low (at least 5 drinks, 2/3 times a week) and high (at least 10 drinks, 3/4 times a week) binge drinking with controls and daily drinkers	Visual Oddball Task	Binge drinkers had reduced N170 amplitude and a difference was also related to the intensity of binge drinking
Folgueira-Ares et al. (2017)	Young adults (N = 50, mean age: 20.6 yo) NIAAA criteria, consumption of at least 3 drinks/hour	Face–Name Pairs Association Task assessing associative memory	No group difference
**VPP – Perceptual and face processing**			

Folgueira-Ares et al. (2017)	Young adults (N = 50, mean age: 20.6 yo) NIAAA criteria, consumption of at least 3 drinks/hour	Face–Name Pairs Association Task	Binge drinkers presented larger VPP amplitude
**N200 – N2a: auditory processing; N2b: attentional resources focusing**			

Maurage et al. (2009)	Young adults (N = 36, mean age: 18.2 yo at first assessment time) Consumption of more than 6 drinks at least once a week	Emotional valence detection task	Binge drinkers had a delayed N2a latency after 9 months of binge drinking
Crego et al. (2009)	Young adults (N = 95, mean age: 18.8 yo) NIAAA criteria, consumption of at least 3 drinks per hour	A visual identical-pairs Continuous Performance Task is a visual task with high working memory load	Binge drinkers had larger N2b amplitude
Maurage et al. (2012)	Young adults (N = 80, mean age: 21.5 yo) Comparison of low (at least 5 drinks, 2/3 times a week) and high (at least 10 drinks, 3/4 times a week) binge drinking with controls and daily drinkers	Visual Oddball Task	Binge drinkers had reduced N2b amplitude and a difference was also related to the intensity of binge drinking
Crego et al. (2012)	Young adults (N = 85, mean age: 21.6 yo) NIAAA criteria, consumption of at least 3 drinks/hour	Visual Oddball Task	No group difference was found for N2b
Petit et al. (2012)	Young adults (N = 36, mean age: 21.6 yo) NIAAA criteria, consumption of at least 3 drinks/hour	Visual Oddball Task with alcohol-related stimuli	No group difference was found for N2b
Watson et al. (2016)	Young adults (N = 50, mean age: 20.6 yo) NIAAA criteria (frequency-based approach to categorize low/high binge drinkers)	Go/No-Go Task with alcohol-related stimuli	No group difference was found for N2b
Park & Kim (2018)	Young adults (N = 50, mean age: 22.2 yo) Consumption of at least 4 (female) or 5 (male) drinks in one occasion more than once in the previous 2 weeks [with minimum 2 (female) or 3 (male) drinks/hour].	Modified spatial 2-back Task assessing working memory	No group difference was found for N2b
**P200 – High level perceptual processing**			

Maurage et al. (2012)	Young adults (N = 80, mean age: 21.5 yo) Comparison of low (at least 5 drinks, 2/3 times a week) and high (at least 10 drinks, 3/4 times a week) binge drinking with controls and daily drinkers	Visual Oddball Task	Binge drinkers had reduced P200 amplitude and a difference was also related to the intensity of binge drinking
**P300 – P3a: attention and stimuli novelty; P3b: closure of cognitive processing**			

Ehlers et al. (2007)	Young adults (N = 125, mean age: 19.9 yo) NIAAA criteria, at least one binge drinking episode during adolescence; with or without drug consumption	Face discrimination Task	Binge drinkers presented reduced P3a latency and reduced P3b amplitude. No difference was observed according to drug consumption
Maurage et al. (2009)	Young adults (N = 36, mean age: 18.2 yo at first assessment time) Consumption of more than 6 drinks at least once a week	Emotional valence detection task	Binge drinkers had a delayed P3b latency after 9 months of binge drinking
Crego et al. (2009)	Young adults (N = 95, mean age: 18.8 yo) NIAAA criteria, consumption of at least 3 drinks/hour	A visual identical-pairs Continuous Performance Task	No group difference was observed for the P3 component
Maurage et al. (2012)	Young adults (N = 80, mean age: 21.5 yo) Comparison of low (at least 5 drinks, 2/3 times a week) and high (at least 10 drinks, 3/4 times a week) binge drinking with controls and daily drinkers	Visual Oddball Task	Binge drinkers presented delayed and reduced P3b as well as longer P3a latency. These difference were also related to the intensity of binge drinking
López-Caneda et al. (2012)	Young adults (N = 48, mean age: 18.7 yo at first assessment time) Consumption of at least 6 drinks once a week or at least 6 drinks (with minimum 3 drinks/hour) once a month	Go/No-Go Task	Binge drinkers had larger P3b during Go trials at baseline and two years later as well as larger P3b during No-Go trials but only after two years of binge drinking.
Crego et al. (2012)	Young adults (N = 85, mean age: 21.6 yo) NIAAA criteria, consumption of at least 3 drinks/hour	Visual Oddball Task	Binge drinkers had increased P3 amplitude
Petit et al. (2012)	Young adults (N = 36, mean: 21.6 yo) NIAAA criteria, consumption of at least 3 drinks per hour	Visual Oddball Task with alcohol-related stimuli	No group difference was found for P3b
Petit et al. (2013)	Young adults (N = 56, mean age: 21.8 yo) Consumption of at least 6 drinks (minimum 3 drinks per hour) at three or four times a week	Visual Oddball Task with alcohol-related and emotional pictures	Binge drinkers had a higher P3b amplitude for the processing of alcohol cues, and this effect was stronger in men
López-Caneda et al. (2013)	Young adults (N = 57, mean age: 18.6 yo, at first assessment time) Consumption of at least 6 drinks once a week or at least 6 drinks (with minimum 3 drinks/hour) once a month	Visual Oddball Task	Binge drinkers showed larger P3b at baseline and this difference increased two years later
López-Caneda et al. (2014b)	Young adults (N = 57), inclusion of an “ex-binge drinkers” group Consumption of at least 6 drinks once a week or at least 6 drinks (with minimum 3 drinks/hour) once a month	Go/No-Go Task	Binge drinkers had larger P3b amplitude during No-Go trials after two years of binge drinking while ex-binge drinkers did not significantly differ from controls and binge drinkers. Consumption speed and age of drinking onset significantly predicted No-Go P3b amplitude
Petit et al. (2014)	Young adults (N = 30, mean age: 22 yo, at first assessment time) Consumption of at least 6 drinks (minimum 3 drinks per hour) at three or four times a week	Visual Oddball Task with alcohol-related and emotional pictures	Binge drinkers had reduced P3b amplitude for the processing of neutral stimuli after one year of binge drinking
Watson et al. (2016)	Young adults (N = 50, mean age: 20.6 yo) NIAAA criteria (approach based on the frequency to categorize low and high binge drinkers)	Go/No-Go Task with alcohol-related stimuli	High and low binge drinkers presented larger P3a amplitude for neutral than alcohol-related stimuli but at different sites (C4 for high binge drinkers and FC2 for low binge drinkers)
Park & Kim (2018)	Young adults (N = 50, mean age: 22.2 yo) Consumption of at least 4 (female) or 5 (male) drinks in one occasion more than once in the previous 2 weeks (with minimum 2 (female) or 3 (male) drinks per hour).	Modified spatial 2-back Task	Binge drinkers showed no significant difference between congruent and incongruent conditions whereas controls presented larger P3a amplitude for congruent stimuli
**LPC – Working memory processing**			

Crego et al. (2010)	Young adults (N = 95, mean age: 18.8 yo) NIAAA criteria, consumption of at least 3 drinks per hour	Identical Pairs Continuous Performance Task	Binge drinkers had a smaller LPC under high working memory load
**Dm effect**			

Folgueira-Ares et al. (2017)	Young adults (N = 50, mean age: 20.6 yo) NIAAA criteria, consumption of at least 3 drinks/hour	Face–Name Pairs Association Task	No Dm effect (larger amplitude for correct than incorrect encoding) was observed in binge drinkers whereas this effect was found in controls

*Note:* VPP: vertex positive potential; LPC: late positive component. Dm effect: Difference due to memory effect; yo = years old. One drink corresponds to one alcohol unit.

### Decrease in electrophysiological activity

Modifications of the P100, N2a, and P3b components were described in the processing of auditory voices in an emotional judgment task, showing delayed latency of these ERP components after 9 months of binge drinking (Maurage et al., 2009); these results thus challenge those of previous studies that proposed that impairments appear only after several years of consumption. At the perceptual level, slower latencies were also observed for P100 and N100 components in an oddball paradigm (a task that compares the brain activations related to repeatedly presented stimuli with those evoked by rare stimuli), and reduced amplitudes were found for N170 and P200 components in binge drinkers (Maurage et al., 2012). This study also emphasized brain dysfunctions in attentional and decisional processes, namely, delayed latencies and reduced amplitudes of N2b and P3b, as well as a longer P3a latency. Furthermore, this study compared two groups of binge drinkers (differing in their intensity, i.e., low and high), a group of daily drinkers (matched on the total number of alcohol doses consumed per week with the low binge drinkers’ group), and a control group. This experimental design has led to important insights by showing a specific effect of the binge drinking pattern, a variation of the brain impairments according to the intensity of binge drinking, and a wide range of cerebral alterations (from early to later stages). Moreover, this amplitude reduction in binge drinkers was confirmed at early (P100) and later (P3b) stages of cognitive processing (Ehlers et al., 2007; Petit et al., 2014). The study by Ehlers et al. (2007) used a facial discrimination task and highlighted a shorter latency for attentional processes related to novelty (P3a) and a reduced amplitude for decisional processes (P3b). The study of Petit and colleagues (2014) confirmed this amplitude reduction for early and later electrophysiological components after 1 year of binge drinking by using a visual oddball task. Compared with control participants, binge drinkers also demonstrated a reduced late positive component under a high working memory load (Crego et al., 2010).

### Increase in electrophysiological activity

Conversely, some studies have indicated enhancement in ERP components. Increased amplitude was mainly observed for later components, as reported by studies with oddball paradigms (Crego et al., 2012; López-Caneda et al., 2013). These studies showed a larger P3b in binge drinkers than in controls. The longitudinal research of López-Caneda and colleagues (2013) confirmed that this difference persisted and was more pronounced after 2 years of continuous binge drinking. During inhibition tasks, a larger amplitude was also found for the P300 component (López-Caneda et al., 2012, 2014b). The first longitudinal study that targeted a binge drinking trajectory showed a higher P300 for Go trials at both evaluation times and a larger P300 for No-Go trials after 2 years of binge drinking (López-Caneda et al., 2012). P300 was detected at frontal, central, and parietal sites and thus corresponded to P3b. Analyses were also conducted to identify the source location, indicating higher activation in the right inferior frontal cortex among binge drinkers during successful inhibition. The second longitudinal study evaluated the persistence of potential cerebral dysfunctions in ex-binge drinkers (López-Caneda et al., 2014b). Findings revealed that binge drinkers had a larger P3b amplitude for No-Go trials than did controls, whereas ex-binge drinkers were in an intermediate position, suggesting that cessation of binge drinking allows recovery of the neurophysiological impairments related to inhibition. Moreover, the study by López-Caneda et al. (2014b) underlined that the P300 amplitude for No-Go trials at the frontal site was predicted by age at drinking onset and consumption speed, supporting the idea that P300 modifications were due to binge drinking. An increased P3 amplitude was also observed during the processing of alcohol cues (Petit et al., 2013) and neutral cues (Watson, Newton-Mora, & Pirkle, 2016), and the effect observed by Petit and colleagues (2013) was particularly patent in male binge drinkers. However, during a working memory task that presented congruent, lure, and incongruent conditions, no difference was observed in binge drinkers at the P3 level, whereas control participants exhibited a larger P3a for congruent stimuli (Park & Kim, 2018). Increased amplitudes were also found for the N2b component in a visual working memory task, which could be explained by a need for higher attentional resources to efficiently perform the task (Crego et al., 2009); however, this result has not been supported by other studies that targeted the N2b component (e.g., Park and Kim, 2018; Petit et al., 2012; Watson et al., 2016).

When investigators explored the effect of alcohol cue processing, a higher P100 amplitude was observed in binge drinkers for the processing of alcohol-related stimuli, regardless of their valence (positive, negative, neutral). This increased P100 was also correlated to longer binge drinking habits and a higher number of doses consumed per occasion (Petit et al., 2012). Another study that evaluated prepotent response inhibition reported a larger amplitude for earlier components (N100) and during the processing of various alcohol-related and neutral stimuli (Watson et al., 2016), particularly in intense binge drinkers (reporting at least eight binge drinking episodes per month). Targeting associative memory processes, Folgueira-Ares et al. (2017) also showed that binge drinkers present an increased amplitude for earlier components, particularly regarding the vertex positive potential, as well as an absence of difference due to memory effect. The converse of this effect was observed in controls, who showed larger amplitudes for correct than for incorrect encoding during the experimental task.

Electrophysiological findings in binge drinking therefore highlight various modifications of brain processing and reveal cerebral dysfunctions even when behavioral performance is preserved. A strong consistency has been observed between studies for the P300 component, which supports the importance of this ERP in alcohol-related disorders (e.g., Ehlers et al., 2007). As a whole, the studies on binge drinking showed a delayed latency for ERP components except for one; the faster P3a latency was interpreted as more rapid detection from theoretical assumptions that the early P300 mainly reflects stimulus detection (Ehlers et al., 2007). However, regarding amplitude, some studies showed reduced amplitude (i.e., fewer resources allocated, and thus impaired processing), while others exhibited increased amplitude (i.e., more resources allocated, and thus possible brain compensation). This dissociation between studies can be understood through the compensatory hypothesis, further explained below.

### Insights from neuroimaging

In line with the section on electrophysiological studies, the current section focuses on functional brain modifications to allow a better understanding of the cerebral correlates of behavioral impairments. Compared with controls, binge drinkers globally present modified brain activity during cognitive tasks. Nevertheless, differential activations can be observed, depending on the nature of the paradigm, the mechanisms involved, and the stimuli presented (e.g., higher activation during inhibition of alcohol-related stimuli, Ames et al., 2014; reduced activation during inhibition of color shapes, Norman et al., 2011). In decision-making tasks, various results appeared, depending on the processes measured (Cservenka & Brumback 2017): a risky decision-making process was associated with reduced activation in the dorsal striatum (Jones, Cservenka & Nagel 2016) and stronger impulsivity was related to reduced activity in the prefrontal dorsolateral cortex (Banca et al., 2016) among binge drinkers. In an anticipated risk-taking task, however, binge drinkers showed higher activities in the prefrontal, orbitofrontal, and upper parietal dorsolateral cortex, associated with risky responses (Worbe et al., 2014). Interestingly, in an affective decision-making context (Iowa Gambling Task), reduced decision-making abilities were observed through increased activities in the left amygdala and bilateral insula in binge drinkers compared with those in controls (Xiao et al., 2013); this observation showed hyperactivity in affective brain systems related to the processing of emotional and rewarding information in the amygdala, as well as to the transformation of physiological signals into feelings in the insula. Enhanced brain activation was also observed during a working memory task in binge drinking, where higher activity in the dorsomedial prefrontal cortex was correlated with the number of drinks consumed on one occasion. Increased activity in the cerebellum, thalamus, and insula was related to the number of drinking occasions per week (Campanella et al., 2013). Moreover, some studies simultaneously showed reduced and increased activity across distinct brain regions in binge drinking (e.g., Schweinsburg et al., 2011; Squeglia et al., 2011), reduced activation being interpreted as a reflection of processing difficulty, whereas increased activation was generally observed in brain regions not related to the ongoing task and thus interpreted as a compensatory activity. In line with this finding, during a verbal learning task, adolescent binge drinkers displayed reduced activity in the inferior frontal and increased activity in the dorsal frontal and parietal brain regions (Schweinsburg et al., 2011). Of note, during working memory tasks, reduced activity was especially found in women and increased activity in men (Squeglia et al., 2011).

### Brief conclusion

Previous sections underlined the cerebral modifications observed in young binge drinkers. Electrophysiological and neuroimaging studies seem to converge, showing distinct brain modifications according to the type of neurocognitive task. Hence, with both tools, inhibition for affective alcohol-related stimuli (Ames et al., 2014; Petit et al., 2012) was associated with increased brain activity. Nonetheless, although it targeted different populations, inhibition during neutral processing led to increased brain activity when the electrophysiological components were explored (e.g., López-Caneda et al., 2012) and reduced brain activity with neuroimaging measures (Norman et al., 2011). Regarding working memory, performance was related to higher brain recruitment in binge drinkers with both neuroimaging and electrophysiological measures (Campanella et al., 2013; Crego et al., 2009), which was interpreted as a compensatory mechanism. Concerning this possible compensation, electrophysiological results focus on the specific process at stake (e.g., attentional component), whereas neuroimaging data contribute to the description of which brain areas/networks are potentially recruited for compensation. Several cognitive functions should still be explored, however, to support the consistency between these techniques (e.g., decision making has to be examined with electrophysiology). In summary, independent of the neuroscience technique used, results have shown that binge drinkers present cerebral modifications of various intensities according to the neurocognitive domain explored. This proposal needs to be confirmed in future studies that target the same processes in populations matched for age and alcohol consumption.

## Questions and perspectives for future research

Overall, this literature review has provided important information about the behavioral and cerebral impairments in executive abilities presented by binge drinkers, as well as possible emotional disturbances recently identified among this population. In view of these results, five key questions are discussed below in terms of the development of this research field: (1) What reorganization occurs in the brain related to binge drinking? (2) What is the causal link between binge drinking and related deficits? (3) Do the deficits persist following changes in alcohol consumption? (4) Is there a transition between binge drinking and alcohol-related disorders? (5) What strategies can prevent or reduce binge drinking habits?

### What reorganization occurs in the brain related to binge drinking?

As previously mentioned, neuroscience studies have emphasized various and sometimes contradictory results (e.g., reduced or increased activations), depending on the experimental paradigm chosen and the type of processing explored. As a potential explanation, increased activation can be interpreted as a compensatory activity, which would allow some behavioral impairments to be overcome. This proposal originated from the observation in neuroimaging research and showed that, during a working memory task, binge drinkers presented reduced cerebral activation in the regions usually involved in this memory processing (e.g., hippocampal regions) and increased activation in other regions (i.e., frontal and motor area), which was not observed in control participants (Campanella et al., 2013; Schweinsburg et al., 2011). This compensatory mechanism would enable binge drinkers to maintain a relatively preserved performance in some behavioral tasks, despite the presence of disturbed brain activities. The same pattern of results was observed during the processing of emotional stimuli (Maurage et al., 2013b). Recently, electrophysiological explorations have also highlighted an enhanced amplitude of ERP components in binge drinkers compared with that of controls, especially for attentional and executive components, which was observed in the absence of behavioral changes (e.g., Crego et al., 2009; López-Caneda et al., 2012, 2014b). This can be explained by increased cerebral recruitment, which compensates for the difficulties associated with specific processing. Binge drinking among young people could therefore be associated with reorganization of brain functioning rather than with global cerebral impairment. Moreover, some behavioral impairments would be undetectable in young adults but could appear during the chronification of excessive alcohol consumption. This proposal of compensatory processes allows an understanding of the cerebral changes observed in binge drinkers and is in line with research showing this compensatory activity in patients recovering from severe alcohol use disorders (e.g., Padilla et al., 2011). Nevertheless, some studies have also highlighted reduced electrophysiological activities in the absence of a behavioral counterpart (e.g., Maurage et al., 2009), raising questions about which impairments are compensated for and how this compensation is indexed. An exploration of the mechanisms implicated in these increased, as opposed to reduced, brain activities appears to be essential for a full understanding of this proposal. According to previous work that suggested compensatory activity in binge drinking (e.g., López-Caneda et al., 2012), such compensation is mostly shown during executive processing (e.g., inhibition). The studies that highlighted reduced electrophysiological activities in the absence of behavioral difficulty (e.g., Ehlers et al., 2007; Maurage et al., 2009, 2012) used experimental paradigms that required fewer executive resources (i.e., detection of emotional faces, oddball paradigm with neutral faces, and valence detection of affective prosody). It might thus be hypothesized that these tasks did not allow identification of behavioral impairments because of reduced involvement of high-level abilities that are impaired in binge drinking, whereas they clearly indicated alterations at the cerebral level. This assumption should be further investigated, however, as other studies with simple paradigms have also shown a larger P3 in binge drinkers (e.g., Crego et al., 2012).

An alternative hypothesis to explain the increased ERP amplitude among binge drinkers may be related to the binge drinking pattern itself. It has been proposed that alcohol withdrawal induces specific cerebral changes observed through hyperexcitability of the central nervous system (i.e., the kindling effect; see Becker, 1998, for a review). This kindling effect is a phenomenon typically observed after repeated weak electrical stimulation of the brain that initially does not produce an effect; repetition leads to the appearance of brain modifications, suggesting sensitization to the stimulation. It has thus been proposed that the repetition of alcohol withdrawal induces a kindling process (Becker, 1998). Current views clearly recognize that binge drinking is characterized by multiple withdrawals (Courtney & Polich, 2009), which leads to brain disturbances in several areas, such as the amygdala or the frontal cortex (Stephens & Duka, 2008). This proposal has been supported by animal studies, showing increased neural excitability following repeated withdrawals (e.g., Duka et al., 2004). Accordingly, Crego and colleagues (2012) proposed that the increased P3b observed in their study could be explained by an imbalance in neural activity due to repeated withdrawal experiences. The difference between reduced and increased amplitude in ERP components might thus be related to the age of binge drinking onset, binge drinking first lowering these ERP amplitudes and then increasing them through the kindling effect in the long run. This hypothesis is consistent with the results of the study by Maurage et al. (2009) that showed reduced P100, N2a, and P3b components after 9 months of binge drinking in youth who did not drink alcohol excessively at baseline. In the same vein, the study of Ehlers et al. (2007) targeted young people who had at least one binge drinking episode in adolescence, which suggests a recent and low binge drinking pattern.

### What is the causal link between binge drinking and related deficits?

The current literature review has emphasized several cognitive and affective impairments in binge drinking, supporting the conclusion that these difficulties are not just observed in severe alcohol use disorders. However, the question of the causal link between these impairments and excessive alcohol consumption remains open. Most of the available work consists of cross-sectional research and does not allow determination of whether these impairments are present before the development of excessive alcohol consumption (i.e., play a causal role in the emergence of binge drinking habits) or result from the alcohol neurotoxic effect associated with binge drinking habits. This question needs to be explored through additional research and literature reviews that focus on adolescents and young adults.

### The presence of vulnerabilities in binge drinkers

Some studies have revealed the presence of impairments or vulnerabilities that have potentially been identified as a cause of binge drinking. These antecedents have been mainly observed in neurophysiological research and would explain the engagement in excessive alcohol consumption in youth (e.g., López-Caneda et al., 2012). More precisely, studies have shown reduced fronto-parietal activations in adolescents prior to any alcohol consumption, and these cerebral activities were identified as predictors of subsequent binge drinking (Norman et al., 2011; Wetherill et al., 2013). Similarly, with participants who were already alcohol consumers, overactivation in the insula, dorsal striatum, ventromedial prefrontal, anterior cingulate, and orbitofrontal cortex during viewing of alcohol cues predicted the transition toward binge drinking 1 year later (Dager et al., 2014).

At the behavioral level, some studies have also addressed this question by recruiting adolescents before the initiation of binge drinking. The results observed are sometimes unexpected; a study that targeted adolescents between 11 and 19 years old and considered four follow-up times showed, for example, no difference regarding executive maturation between participants who remained teetotalers and those who initiated binge drinking habits (Boelema et al., 2016). These findings can be related to the difficulty in detecting behavioral differences at this early stage, combined with a need to use basic tasks for the longitudinal observation of executive maturation. In working with adolescents, Gil-Hernandez et al. (2017) suggested that compensatory mechanisms may exist in the adolescent brain that allow young people to achieve a performance similar to that of their non-binge-drinking peers at the behavioral level. This study showed executive difficulties in binge drinkers compared with controls only in a group of older adolescents and young adults (19–22 years), thus proposing that behavioral impairments emerge only at this later stage of binge drinking habits. Among university students who had already initiated alcohol consumption, only risky decision making and not inhibitory control predicted the binge drinking score 18 months later (Bø et al., 2017). However, this study adopted a continuous approach to alcohol consumption (i.e., targeted the binge drinking score and did not propose group comparisons), and thus did not allow exploration of hazardous binge drinking. Moreover, no information was collected about potential changes in the performance of these participants at follow-up, thus preventing definitive conclusions regarding the causal link.

### Behavioral and cerebral impairments as a consequence of binge drinking

Some studies have also supported the inverse relationship and have proposed that the impairments observed in binge drinkers are the direct consequence of alcohol consumption. One study that targeted adolescent low drinkers indicated that alcohol consumption 4 years later (mean = 54 g per week at follow-up when participants were 18 years old) was not predicted by cognitive performance (inhibitory control and shifting) or brain activation (Jurk et al., 2016), suggesting that both cognitive impairments and brain modifications are the consequences of alcohol consumption rather than its cause.

First, this proposal is supported by neuroimaging studies that showed that binge drinking predicted cerebral alterations. In particular, a higher number of drinks consumed predicted smaller left hemisphere cerebellar white and gray matter, as well as reduced right hemisphere cerebellar gray matter volume (Lisdahl et al., 2013). Research also implicated gender, describing differential brain modifications in men and women (e.g., Squeglia et al., 2012). This proposal that impairments are the consequences of binge drinking is supported more clearly by longitudinal research. The study of Maurage and colleagues (2009) identified a slowing down of cerebral activity during the processing of affective voices after 9 months of binge drinking among participants who had similar brain functioning to that of controls at baseline. Moreover, in a follow-up study of 8 years from adolescence to adulthood, it appeared that both binge drinking and a family history of severe alcohol use disorders were associated with alterations in the developmental trajectory of impulsivity, reflected as difficulty in delaying a reward in binge drinkers with a family history of alcohol-related disorders (Jones, Steele & Nagel 2017). This study emphasized a similar performance at baseline between future binge drinkers and controls. Only adolescents with a family history of alcoholism were impaired in impulsive choices at first assessment. Interestingly, adolescents with a family history of alcohol use disorders who did not engage in binge drinking did not present impairments at follow-up, whereas those who engaged in binge drinking showed stronger impairments. While targeting an at-risk population, namely, individuals with a family history of severe alcohol use disorders, this study confirms the proposal of previous studies by indicating that the difficulty in delaying a reward is in particular strongly associated with binge drinking and is related to the number of alcohol doses consumed.

Second, the assumption that alterations constitute the consequences of binge drinking is also in accordance with the proposal of Stephens and Duka (2008), who conveyed that specific alternations between alcohol intoxication and withdrawal in binge drinking induce changes in neuronal plasticity and result in cognitive and emotional impairments. This research encompasses several studies conducted in patients with severe alcohol use disorders and in social drinkers who had binge drinking episodes (see Duka et al., 2004, for more details). It showed both executive impairments and altered conditioned responses, potentially related to dysfunctions in the frontal regions and amygdala, and directly related to the number of withdrawals. Notably, to test the proposal that behavioral and brain impairments were associated with alcohol consumption, studies were conducted that involved animals exposed to specific alternations between intense drinking and withdrawal. Findings supported that binge drinking leads to impaired functioning in the frontal cortices and amygdala (Stephens & Duka 2008), leading to neurodegeneration similar to that seen in severe alcohol use disorders. In human populations, this deleterious effect seems particularly important in adolescents or people with genetic risk factors (see Crews, 2008, for a review).

In summary, on the basis of animal and human studies, it can be proposed that impairments observed in young drinkers might simultaneously result from binge drinking and also be involved in the emergence of chronic alcohol abuse. Investigators clearly identified that frontal impairments facilitate the subsequent loss of control in alcohol consumption and underlined the possible difficulties in processing emotional events because of amygdala dysfunctions. Finally, this assumption of specific impairments due to the alternation between intoxications and withdrawals is supported by a study by Dingwall, Maruff, and Cairney (2011) that showed that binge drinking was related to impairments and patterns of recovery similar to those found in individuals with severe alcohol use disorder. This study targeted people involved in therapeutic programs for excessive alcohol use and compared binge drinkers (drinking at least six alcohol doses per occasion, less than four times a week), individuals with severe alcohol use disorder (drinking at least six alcohol doses per occasion, more than four times a week), and controls. Results showed that both groups of alcohol users presented impairments in visuo-motor and learning skills, as well as in memory and executive functions. While visuo-motor difficulties persisted in the long term (i.e., at 11 months follow-up) in the two groups of drinkers, recovery was observed for other processes within the four detoxification weeks. Although it did not focus on the specific relationship with withdrawal episodes, this study suggests that alcohol alters cognitive and motor functioning in a similar way to that in excessive episodic or chronic drinking patterns.

As a whole, it is clear that the neurotoxic effects of alcohol, and potentially the specific alternation between intoxication and withdrawal, induce brain damage, resulting in impairments among binge drinkers. Most studies in the current literature support such a causal direction. However, it also appears that some factors, present before the start of alcohol consumption, can predispose an individual to engage in excessive alcohol consumption (e.g., modification in executive brain networks). This effect was mainly investigated in neuroscience studies, but future work should specify these possible predispositions with combined behavioral and cerebral measures and target the mechanisms involved with sufficiently complex paradigms. Complementary investigations of the underlying mechanisms appear to be crucial for a better understanding of the disrupted functioning and causal relationships in binge drinking.

### Do the deficits persist following changes in alcohol consumption?

An important question relates to the persistence of these impairments when binge drinking becomes less frequent, as it is usually observed in adulthood. Animal studies suggest potential brain recovery after binge drinking (Crews, 2008), but studies with young human drinkers are lacking and remain uncertain regarding the temporal framework and differential recovery across cognitive processes. Longitudinal studies that focus on university students have shown that most binge drinkers maintain their excessive drinking habits throughout their university years, despite the associated negative consequences (e.g., unwanted sexual relationships or violent behaviors; Martinez, Sher & Wood 2014). However, it has been reported that, generally, binge drinking decreases after university years, as supported by a 9-year follow-up study (Gómez et al., 2017). People who were engaged in binge drinking but who return to stable alcohol consumption can still present behavioral impairments in specific cognitive abilities such as verbal memory (Mota et al., 2013), but recover some brain processes such as those involved in inhibition performance (López-Caneda et al., 2014b). Recent studies have also provided information about the evolution of memory and decision-making processes over time. A 6-year longitudinal study on binge drinking in late adolescence measured logical memory (Wechsler Memory Scale) in stable binge drinkers, non-binge drinkers, and ex-binge drinkers. Results showed that, compared with non-binge drinkers, binge drinkers presented impaired immediate and delayed recall. Interestingly, in the midterm (after about 2 years), ex-binge drinkers showed difficulties in immediate and delayed recall but these impairments disappeared in the long term (Carbia et al., 2017a). This study is interesting because it extends the time frame of evaluation described by previous studies. However, as frequently found in this type of longitudinal evaluation, there was a strong experimental mortality in the successive steps, resulting in a poor final sample size in some subgroups at follow-up (e.g., n = 4 for the stable binge drinkers’ group at the third assessment). Replications of these results are thus necessary to support the absence of impairments in ex-binge drinkers in the long term. Moreover, within a 4-year period, a study that compared the same subgroups but evaluated decision making through the Iowa Gambling Task showed no decision-making impairment in stable binge drinkers or ex-binge drinkers (Carbia et al., 2017b). Therefore, research on the persistence of impairments associated with binge drinking should be extended. Moreover, although in this section we focused on behavioral performance, it has been shown that some impairments are not detectable at the behavioral level and that they could be partially compensated for at the cerebral level. Accordingly, it can be hypothesized that the perpetuation of binge drinking could lead to more pronounced behavioral impairments that can no longer be compensated for. Conversely, it can also be suggested that, if alcohol consumption is regulated with time, these processes could recover, as observed in the study of López-Caneda and colleagues (2012). Notably, the recovery hypothesis is supported by research conducted in severe alcohol use disorders that showed recovery in cognitive abilities with the maintenance of early abstinence (Munro, Saxton, & Butters, 2000) but, surprisingly, with no complementary beneficial effect of long-term abstinence (Fein et al., 2006). Nevertheless, recovery does not appear to be total, as patients with long-term abstinence still show persistent impairments in inhibitory and attentional control (Naim-Feil et al., 2014), although, consistent with the findings reported by Fein et al. (2006), abstinence duration was not related to cognitive performance. These findings in the context of severe alcohol use disorders thus align nicely with the preliminary results observed in binge drinking, showing partial recovery with reduced alcohol consumption but persistence of some impairments in the midterm.

### Is there a transition between binge drinking and alcohol-related disorders?

The continuum hypothesis, as is often described in the literature for severe alcohol use disorders, has been used to characterize the links between binge drinking and other alcohol consumption patterns in two distinct ways. The first concerns the continuum of alcohol consumption patterns and proposes that binge drinkers are more at risk for developing severe alcohol use disorders than are light or moderate drinkers (e.g., Bonomo et al., 2004). The second refers to the continuum of the impairments observed in the two patterns of alcohol consumption and suggests that binge drinkers present qualitative impairments similar to those of patients with severe alcohol use disorders (e.g., Sanhueza, García-Moreno & Expósito 2011), regardless of the possible evolution of the drinking pattern (i.e., development of severe alcohol use disorders).

### The continuum between consumption patterns

From the very start of research on binge drinking, several authors observed that binge drinking is related to important negative consequences (e.g., Wechsler et al., 1994), potentially leading binge drinkers to be more at risk for severe alcohol use disorders. This was later confirmed by a large exploration of students from 119 colleges and universities, indicating that binge drinkers were far more likely (i.e., 19 times higher odds) to develop severe alcohol use disorders later in life than were non-binge drinkers (Knight et al., 2002). This proposal has moreover been tested in cohort studies over 6, 10, and 14 years, showing that binge drinking in adolescents preceded (Bonomo et al., 2004) and predicted (Jennison, 2004; Viner & Taylor 2007) severe alcohol use disorders in young adulthood. In addition, research with alcohol-dependent patients revealed that almost half of them were binge drinkers in late adolescence (Enoch, 2006). The continuum hypothesis thus postulates that binge drinking and severe alcohol use disorders are two successive steps of the same phenomenon, possibly characterized by analogous qualitative impairments at behavioral and brain levels. Therefore, these two disorders could be described as a linear worsening of cognitive and affective abilities that promote the maintenance of excessive drinking habits and the possible transition toward severe alcohol use disorders.

### The continuum of impairments

The proposal of similar qualitative impairments between binge drinking and severe alcohol use disorders was supported by studies that suggested that the specific alternation between intense intake and withdrawal, which characterizes binge drinking, could lead to consequences similar to those of severe alcohol use disorders at cognitive and emotional levels (Stephens & Duka 2008). Some studies have observed similarities in the impairments found among binge drinkers and individuals with severe alcohol use disorders for cognitive processes such as attention, memory, and executive functions (Goudriaan et al., 2007; Hartley et al., 2004), as well as for cerebral functioning (e.g., López-Caneda et al., 2017; Maurage et al., 2013b). While this hypothesis has not received direct experimental support from comparisons between binge drinkers and patients with severe alcohol use disorders, an investigation of executive functioning showed that young adult binge drinkers had a similar performance to that of elderly adults (Sanhueza et al., 2011), thus conveying the proposal of premature aging in individuals engaged in binge drinking. These findings reinforce the continuum hypothesis by showing that the specific binge drinking pattern leads to strong impairments in critical processes implicated in addiction, namely, executive functions (Crews & Boettiger 2009). Globally, these findings confirm that binge drinking has deleterious consequences at several levels and could belong to the whole spectrum of alcohol-related disorders, in particular because of the alternations between intoxication and withdrawal.

To develop further insights into this question, some studies have been conducted on binge drinking with paradigms that had already been used among patients who had severe alcohol use disorders, thus allowing indirect but interesting comparisons. Findings globally underline that binge drinkers are characterized by various impairments, supporting the presence of alterations that are comparable to those observed in severe alcohol use disorders. Nevertheless, these findings emphasize that the difficulties found in binge drinkers are related to specific processes:

- Performance monitoring difficulties but no global inhibition alterations (Bø et al., 2016a; Lannoy et al., 2017b; Lannoy et al., 2018a; Smith & Mattick, 2013).
- Alerting and executive control of attention but no general attentional impairment (Hartley et al., 2004; Lannoy et al., 2017a; Sanchez-Roige et al., 2014).
- Emotional recognition only for complex morphed stimuli (Lannoy et al., 2018b).
- Cerebral modifications during emotional processing, but no strong behavioral impairment (Huang et al., 2017; Maurage et al., 2009), notably for cross-modal integration (Lannoy et al., 2018c).

In severe alcohol use disorders, more global deficits were found, such as general executive impairment (Brion et al., 2017a), behavioral (Maurage et al., 2007) and cerebral (Maurage et al., 2013c; Maurage et al., 2008a) markers of impaired emotional cross-modal integration, and altered inhibition, notably during the processing of alcohol cues (e.g., Noël et al., 2007).

Possible similarities between binge drinkers and patients with severe alcohol use disorders were thus observed for lower level cognitive abilities, electrophysiological processing of error detection (performance monitoring), and some emotional abilities. Regarding attentional networks, the same paradigm has been used in binge drinking and severe alcohol use disorders, and the results were similar, showing specific difficulty in executive control of attention (Lannoy et al., 2017a; Maurage et al., 2014). However, binge drinking was also characterized by an impaired alerting network (Lannoy et al., 2017a). Regarding this result, whereas binge drinkers were asked to abstain from alcohol during the 3 days before the experiment, participants were all engaged in a stable binge drinking pattern and patients with severe alcohol use disorders were recruited during their third week of detoxification (Maurage et al., 2014), which could potentially explain the difference in the alerting component. At the electrophysiological level, comparable results were observed through an enhanced Error-Related Negativity in abstinent patients with severe alcohol use disorders (Padilla et al., 2011) and binge drinkers (Lannoy et al., 2017b), indexed as compensatory activity. Finally, regarding emotional processing, poorer emotional abilities were found for the processing of various emotions, both in severe alcohol use disorders (e.g., Kornreich, 2002; Maurage et al., 2011) and binge drinking (Lannoy et al., 2018b). Moreover, while binge drinkers presented cerebral reorganization rather than clear impairments (e.g., Huang et al., 2017; Lannoy et al., 2018c), comparable difficulties were found in binge drinking and severe alcohol use disorders: disrupted cross-modal integration, differential processing for anger (Maurage et al., 2008a; Maurage et al., 2008b), and specific difficulty in processing incongruent cross-modal trials (Brion et al., 2017b). Table 3 summarizes the similarities and dissimilarities between binge drinking and severe alcohol use disorders regarding the cognitive functions explored in this paper.

**Table 3:** Comparison between binge drinking and severe alcohol use disorders.

Cognitive function Sub-components	Binge drinking	Severe alcohol use disorders	Comparison

**Attentional abilities**			

Alerting	*Impaired*	Preserved	x
Orienting	Preserved	Preserved	V
Executive control	*Impaired*	*Impaired*	V
**Executive abilities**			

Shifting	Preserved	*Impaired*	X
Updating	Preserved	*Impaired*	X
Inhibition	*Impaired*	*Impaired*	v
Error-monitoring	*Modified*	*Modified*	V
**Emotional abilities**			

Emotional decoding	*Impaired*	*Impaired*	v
Anger processing	*Modified*	*Impaired*	v
Happiness processing	Preserved	Preserved	V
**Crossmodal abilities**			

Crossmodal integration	Preserved	*Impaired*	X
Congruent stimuli processing	*Modified*	*Impaired*	v
Incongruent stimuli processing	*Modified*	*Impaired*	v

*Legend:* V = comparable, v = potentially comparable, needs further investigation, X = different, x = potentially different, needs further investigation.

As a whole, mixed results are currently reported regarding the continuum hypothesis, several studies showing qualitatively similar impairments in the targeted processes, but also indicating differential and more specific underlying mechanisms among binge drinkers. First, the presence of more pronounced impairments in severe alcohol use disorders can be explained by the repeated impact of alcohol consumption (Crews, 2008), as patients were drinking alcohol excessively for a long period and were potentially ex-binge drinkers (Enoch, 2006). Moreover, the continuum hypothesis cannot be discussed without taking into account the recent findings that show heterogeneity in binge drinking profiles (Gierski et al., 2017; Lannoy et al., 2017d). These results underline that binge drinkers are not a unitary group, showing that some students are instead involved in a recreational pattern of consumption, while others are clearly identified as being implicated in a more dangerous alcohol consumption pattern. Accordingly, it could be hypothesized that intense binge drinkers persist in excessive alcohol consumption and develop cognitive and emotional difficulties, which lead them to poorly regulate their drinking behaviors and therefore evolve toward severe alcohol use disorder, as has been underlined in previous longitudinal findings (e.g., Bonomo et al., 2004). Three main research perspectives might thus be developed for future research:

1. The exploration of cognitive and affective impairments in different subgroups of binge drinkers, differing in alcohol consumption patterns. This subgroup comparison could be based on variation in binge drinking criteria from cluster analysis and would allow the targeting of profiles of at-risk binge drinkers.
2. A direct exploration of similarities and differences between binge drinking and severe alcohol use disorders to compare, beyond the continuum of consumption patterns, the variation of existing impairments. This exploration could be made through the comparison between binge drinkers, recently detoxified patients, and matched control participants. This design would allow determination of the position of binge drinkers compared with a control group of young moderate drinkers, a control group of older but healthy participants with moderate alcohol consumption, and a group of patients with severe alcohol use disorders.
3. In line with previous studies (e.g., Bonomo et al., 2004; Jennison, 2004), longitudinal studies with multiple testing sessions starting during early adolescence to explore the mutual influences between binge drinking habits, cognitive impairments, and the potential appearance of severe alcohol use disorders.

### What strategies can prevent and reduce binge drinking habits?

Several interventions have been proposed to prevent binge drinking and, more globally, excessive alcohol consumption among young people. In this section, we briefly describe the interventions that aim to reduce alcohol consumption and to avoid the emergence of binge drinking habits. Specific tools to rehabilitate the cognitive processes impaired in binge drinking, particularly inhibitory control, are then addressed.

Different strategies to reduce alcohol consumption have shown promising results. First, motivational intervention aims to increase the intrinsic motivation to change through ambivalence exploration and resolution (Yurasek et al., 2015). This tool is particularly useful with binge drinkers, as they usually perceive few negative consequences associated with their consumption. Second, intention implementation aims to help individuals to learn a new and adapted response when confronted with situations previously associated with a maladaptive one, such as excessive alcohol consumption. Results showed that students who took part in an intention implementation program reported reduced alcohol consumption and less binge drinking behavior 1 week later (Norman & Wrona-Clarke, 2016). Expectancy challenge is mainly used with adolescents and aims to modify dysfunctional expectancies toward alcohol by proposing that participants drink a beverage (alcohol or placebo) without knowing its content and then engage in social activities with other adolescents. A meta-analysis showed effects of expectancy challenge but mainly in the short term, with no maintenance at 4-week follow-up (Scott-Sheldon et al., 2012). These strategies have therefore demonstrated their potential efficiency, but results should be confirmed through longer follow-up periods.

In addition to preventive intervention, impaired cognitive processes can be targeted in rehabilitation programs. Inhibition training for alcohol-related cues has been proposed, mostly through a Go/No-Go task with a No-Go alcohol condition in which participants learn to inhibit their motor response when confronted with alcohol-related cues (Houben et al., 2011). The study evaluated the impact of such training on alcohol consumption immediately after the task in a real situation and with self-reported measures in the following week. After inhibition training for beer cues, hazardous drinkers presented decreased alcohol consumption (both in free drinking following the task and in their weekly use). Moreover, conversely, participants in the control condition (“Go” alcohol) reported increased alcohol consumption in the following week. Nevertheless, proposing the same paradigm in two sessions, and comparing results with those of an active control group (i.e., online personalized feedback based on alcohol consumption), Bowley and colleagues (2013) were not able to confirm these results. They indicated that participants in both active training groups (feedback intervention and inhibition training) drank less, but only in the test presented after the training, with no significant difference in follow-up self-reported alcohol consumption (Bowley et al., 2013). These results were also reported in a recent study that compared inhibition training for alcohol cues and alcohol approach modification training (Di Lemma & Field, 2017). Both interventions appeared to have the same efficacy in laboratory settings, but these effects did not extend to real-life situations. These findings thus show preliminary interesting results, but there is a clear need for future work before a conclusion can be drawn regarding the clinical efficiency of such programs in binge drinking.

Finally, our review paper underlines some brain modifications and the presence of difficulties even in the absence of behavioral impairments. From this perspective, there is also a new line of research that focuses on neuroscience-based intervention techniques such as neuromodulation [e.g., repeated transcranial magnetic stimulation and transcranial direct current stimulation (tDCS)]. These noninvasive techniques allow stimulation of specific brain areas to improve targeted cognitive abilities and promote compensation for some difficulties. Studies with binge drinkers showed craving reduction after tDCS stimulation of the left dorsolateral prefrontal cortex during simple reading (den Uyl, Gladwin, & Wiers, 2015), but no effect was found on implicit associations and alcohol approach tendencies. This research thus represents an interesting avenue, but the specific effects still need to be determined. To this end, ERP could be useful in enabling the exploration, with high temporal resolution, of the specific mechanisms modulated following transcranial stimulation (Miniussi, Brignani, & Pellicciari, 2012). With healthy participants, tDCS was applied to the right inferior frontal cortex during inhibition of prepotent response (Campanella et al., 2017). The same inhibition task and another control task (facial detection) was then performed with an electroencephalogram recording, allowing the observation of N2 and P3 components from the subtraction between Go and No-Go trials. Results indicated no significant difference at the behavioral level between tDCS and sham conditions. However, a reduction in electrophysiological activity related to the P3 component was found, suggesting that tDCS allowed a decrease in the resources recruited to efficiently perform the task (Campanella et al., 2017).

## Conclusion

This review paper offered an overview of the literature on the executive and emotional impairments found in binge drinkers by describing behavioral, electrophysiological, and neuroimaging studies, as well as by critically discussing five key issues identified in this research field. In particular, we emphasized that the impairments observed in binge drinkers are mainly related to inhibition and sometimes not indexed at the behavioral level but detectable through cerebral modifications. Moreover, difficulties in processing emotional content are also detected at both the behavioral and the neurophysiological level. From these findings, we have attempted to offer a better understanding of the cerebral deficits observed in binge drinkers by comprehensively describing the compensatory hypothesis and the kindling effect. We have also questioned the causal relationship between excessive alcohol use and cognitive/affective impairments or cerebral modifications, as well as highlighting that the binge drinking pattern might constitute a specific risk factor for these impairments. On the other hand, some predispositions could exist and should be taken into account in future studies. We then reviewed the few studies that have explored whether deficits persist after binge drinking cessation and proposed that, although encouraging results have been observed, there is an urgent need to comprehensively investigate this question. Next, we discussed the continuum hypothesis, suggesting a link between binge drinking and severe alcohol use disorders. We have shown that, while these two alcohol consumption patterns share several common features, binge drinking is associated with more limited and specific impairments. Concrete proposals to further answer this question, which constitutes an important topic for both fundamental and clinical purposes, have also been suggested. Finally, we described clinical perspectives addressed through prevention and intervention strategies. Overall, promising results have been shown, but they should be further specified, explored in greater depth, and evaluated by using longer follow-up periods.
